# Pyruvate: A key Nutrient in Hypersaline Environments?

**DOI:** 10.3390/microorganisms3030407

**Published:** 2015-08-07

**Authors:** Aharon Oren

**Affiliations:** Department of Plant and Environmental Sciences, The Alexander Silberman Institute of Life Sciences, The Hebrew University of Jerusalem, Edmond J. Safra Campus, Jerusalem 91904, Israel; E-Mail: aharon.oren@mail.huji.ac.il; Tel.: +972-2-6584-951; Fax: +972-2-6584-425

**Keywords:** pyruvate, *Halobacteriaceae*, *Haloquadratum*, *Halosimplex*, *Spiribacter*, glycerol, dihydroxyacetone

## Abstract

Some of the most commonly occurring but difficult to isolate halophilic prokaryotes, Archaea as well as Bacteria, require or prefer pyruvate as carbon and energy source. The most efficient media for the enumeration and isolation of heterotrophic prokaryotes from natural environments, from freshwater to hypersaline, including the widely used R2A agar medium, contain pyruvate as a key ingredient. Examples of pyruvate-loving halophiles are the square, extremely halophilic archaeon *Haloquadratum walsbyi* and the halophilic gammaproteobacterium *Spiribacter salinus*. However, surprisingly little is known about the availability of pyruvate in natural environments and about the way it enters the cell. Some halophilic Archaea (*Halorubrum saccharovorum*, *Haloarcula* spp.) partially convert sugars and glycerol to pyruvate and other acids (acetate, lactate) which are excreted to the medium. Pyruvate formation from glycerol was also shown during a bloom of halophilic Archaea in the Dead Sea. However, no pyruvate transporters were yet identified in the genomes of halophilic Archaea, and altogether, our understanding of pyruvate transport in the prokaryote world is very limited. Therefore, the preference for pyruvate by fastidious and often elusive halophiles and the empirically proven enhanced colony recovery on agar media containing pyruvate are still poorly understood.

## 1. Introduction

Attempts to enumerate heterotrophic prokaryotes in hypersaline brines approaching salt saturation, such as those found in saltern crystallizer ponds and in some natural salt lakes, by counting colonies on agar plates invariably yield counts at least one or two orders of magnitude below the number of cells observed microscopically. This “great plate count anomaly” is a general phenomenon and is by no means restricted to high-salt ecosystems. For most environments, some of the numerically most important types still have not been brought into culture. Using a diversity of solid media and incubation times of two months and longer, some improvement can be achieved; most groups of halophilic Archaea (class *Halobacteria*) encountered in a 16S rRNA gene library from an Australian saltern crystallizer pond were found to be cultivable [1].

A major breakthrough in our understanding of the microbiology of neutral-pH, salt-saturated brines was achieved in 2004 with the isolation of the flat square Archaea that can form 70%–80% or more of the prokaryotes inhabiting some salterns [2,3]. This organism has been described as *Haloquadratum walsbyi* [4]. The growth media used for the isolation of this intriguing and elusive organism all contained pyruvate, a compound that had not been included in the standard media used for the cultivation of extremely halophilic prokaryotes for nearly a century. Pyruvate was also a key compound in growth media for the halophilic gammaproteobacterium *Spiribacter salinus*, an organism recently recognized as a significant component of the saltern microbial community at intermediate salinities on the basis of metagenomics studies [5,6], and in media for the cultivation of *Halosimplex carlsbadense*, an atypical halophilic archaeon isolated from a salt mine [7].

Here I review the currently available information on pyruvate as a key compound in growth media for the study of heterotrophic halophilic communities, including questions about the possible availability of the compound to halophilic Archaea and Bacteria in nature and about the ways pyruvate is taken up and metabolized by these organisms.

## 2. Pyruvate As a Key Compound for the Nutrition of Selected Halophilic Microorganisms

In the proposed minimal standards for the description of new taxa in the class *Halobacteria*, endorsed by the Subcommittee on the Taxonomy of *Halobacteriaceae* of the International Committee on Systematics of Prokaryotes [8], pyruvate is one of the compounds included in the list of substrates recommended to be tested during the characterization of new taxa of aerobic halophilic Archaea. In most such species descriptions published in recent years, growth on pyruvate as single carbon source and or growth stimulation by pyruvate in complex media was indeed tested. Pyruvate was included in the tests performed for 33 out of 41 species descriptions published in 2013–2014. Nearly all were reported to be able to grow on pyruvate as the sole carbon and energy source and/or showed growth stimulation by pyruvate. There were two exceptions: *Halapricum salinum*, an organism that uses a few sugars but is not stimulated by pyruvate, glycerol, lactate, and acetate [9] and *Halarchaeum nitratireducens*, a species that uses a few sugars as well as glycerol but none of the organic acids or amino acids tested with the exception of succinate [10]. Whether the lack of use of organic acids by this moderately acidophilic organism (pH range 4.2–6.0, optimum growth at pH 5.2–5.5) may be connected to the toxicity of unionized acids at low pH remains to be ascertained.

Two species of halophilic Archaea appear to be dependent on pyruvate as a key component in their growth media in the laboratory: *Hsx. carlsbadense* and *Hqr. walsbyi* [2,3,4,7,11]. Pyruvate as the preferred nutrient for the cultivation of certain fastidious halophilic prokaryotes was first documented in the case of *Hsx. carlsbadense*, isolated from Permian rock salt collected from a salt mine in New Mexico, USA. In contrast to most other members of the *Halobacteria*, this species does not grow on rich complex media, but it can only be cultured in defined media with pyruvate, glycerol, glycerol and acetate, or glycerol and pyruvate as sole carbon and energy sources; however, pyruvate and acetate did not support growth [7]. Two additional new species were recently described in the genus *Halosimplex*: *Hsx. pelagicum* and *Hsx. rubrum*, both isolated from salted brown algae of the genus *Laminaria*. An oligotrophic medium with low concentrations of yeast extract and fish peptone, supplemented with pyruvate at concentrations of 0.05–1.0 g/L, served for their isolation. The two new *Halosimplex* species can grow on pyruvate, but their range of compounds used is wider than that for *Hsx. carlsbadense*, and includes several sugars, amino acids, and organic acids [12].

Isolation of the square archaeon now known as *Hqr. walsbyi* was reported independently by two groups in 2004. Bolhuis *et al.* [3] retrieved the organism from a saltern in Spain. They used a medium containing low concentrations of yeast extract supplemented with pyruvate, acetate, or lactate, reported earlier as possible products formed by other halophilic Archaea in hypersaline brines from glycerol, the osmotic solute produced in hypersaline environments by algae of the genus *Dunaliella* [13], as further discussed below. Burns *et al.* [2] obtained a nearly identical isolate from a saltern in Australia by use of media that contained brine from the saltern, 5 g/L pyruvate, and 50 μM amino acids. In the species description paper, the authors recommended media containing 10 mM pyruvate, 0.25 g/L peptone (Oxoid), and 0.05 g/L yeast extract. Pyruvate is the best carbon source for growth of *Hqr. walsbyi*; no growth enhancement was obtained in the presence of acetate and other organic acids, glucose, and other sugars, glycerol, or amino acids [4].

An entirely different type of halophile that uses pyruvate as its preferred substrate is the moderately halophilic (growth range 100–250 g/L salt with an optimum at 150 g/L) gammaproteobacterium *Spiribacter salinus*. Existence of this organism, which can be abundantly present in saltern evaporation ponds of intermediate salinity, was first recognized during the analysis of the metagenome of a Spanish saltern. It was isolated on a medium containing 1.1 g/L pyruvic acid and 1 g/L yeast extract as the sole nutrients. For routine cultivation, a medium with 1.1 g/L pyruvic acid, 2.5 g/L yeast extract, and 1 g/L glucose was recommended. It grows on pyruvate or glycerol as carbon and energy sources; many other simple compounds, including common sugars, organic acids, and amino acids, are not used [5,6].

## 3. Modified R2A Agar for the Recovery of Colonies of Halophiles

Attempts in the past to improve the recovery of bacterial colonies from oligotrophic waters resulted in the formulation of the medium known as R2A agar. It is a medium with relatively low nutrient concentrations, and it contains the following organic components: 0.5 g/L glucose, 0.5 g/L starch, 0.3 g/L Na-pyruvate, 0.5 g/L proteose peptone, 0.5 g/L casamino acids, and 0.5 g/L yeast extract [14]. Pyruvate is thus one of the constitutents. The developers of the medium did not provide a rationale for why pyruvate was included beyond the statement that the medium contains a greater variety of nutrients than other conventional media.

R2A agar is now widely used in ecological studies of natural waters and soils because of the (relatively) high colony counts obtained and the possibility to isolate organisms that are not readily recovered on the commonly used rich media. The medium was also adapted to the recovery of halophiles from saltern brines and sediments by the addition of high concentrations of salts reflecting the salinity of the environment investigated. Such modified R2A media were used in ecological studies of saltern pond brines in Israel, Australia, and the USA [1,15,16,17]. For the oligotrophic saltern at Shark Bay, Australia, R2A-based media with 3.3 g/L K-acetate and 1 g/L pyruvic acid gave the highest counts of different media tested [15]. Colony counts of saltern crystallizer brine from Eilat, Israel were up to 1.4 × 10^5^ per mL. This is still significantly less than the total microscopic count of 1.3 × 10^8^ per mL reported for the sample investigated, but other media tested lacking pyruvate yielded 1–2 orders of magitude fewer colonies than the R2A-based medium [15]. Also, for the San Francisco Bay salterns, an R2A-based medium with 0.5 g/L glucose, 0.5 g/L starch, and 0.3 g/L pyruvate enabled recovery of the highest numbers of both total colonies and red-pigmented colonies [16]. For an Australian saltern (Geelong, Victoria), relatively high viable counts (~10^6^/mL, compared to microscopic counts of 1.2 ± 0.2 × 10^7^ per mL) were obtained on solidified media after long (>8 weeks) incubation times on media that contained 50 mM each of glycerol, glycolic acid, pyruvate, lactate, and acetate; however, this medium was not clearly superior to four other minimal media tested that did not contain pyruvate, or to a richer medium [1]. High-salt agar media based on the R2A agar recipe were also used to recover colonies of cultivable halophilic Archaea from the surface sediments of solar salterns in Tunisia [18].

## 4. Pyruvate Transport in Halophilic and in Non-Halophilic Prokaryotes

The intracellular metabolism of pyruvate in halophilic Archaea, including conversion to acetyl-CoA, oxalate, and alanine, is relatively well known [19], but we have little information about the way the compound is transported into the cell. ^13^C-labeled pyruvate was shown to be taken up by *Halobacterium salinarum* [20], but the mechanism was not further elucidated. In *Hqr. walsbyi*, pyruvate probably enters the cell by diffusion since specific uptake systems have not been identified in the genome. The same is the case for glycerol [21]. Also, in the two genomes of *Spiribacter* spp., no pyruvate transporters could be annotated [6].

Surprisingly, little is also known about the mechanisms of pyruvate uptake by non-halophilic prokaryotes. A study from the 1970s showed that *Escherichia coli* membrane vesicles transport pyruvate when energized by ascorbate-phenazine methosulfate, but no such transport could be demonstrated in membrane vesicles of *Bacillus subtilis* and of a *Pseudomonas* sp. [22]. An inducible (Usp) and a constitutive (PrvT) transport system were recently characterized in *E. coli* K12 [23]. *Corynebacterium glutamicum* contains MctC, a carrier for acetate and propionate that depends on the electrochemical proton potential and has a low affinity for pyruvate. Putative transport systems similar to MctC of *C. glutamicum* were found in other *Corynebacterium* spp., in *Streptomyces coelicolor*, in *Mycobacterium smegmatis*, in *Nocardia farcinica*, in *E. coli*, and in *B. subtilis* [24]. *Rhizobium leguminosarum* has a monocarboxylate permease MctP, a sodium-solute transporter that transports lactate (K_M_ 4.4 μM) and pyruvate (K_M_ 3.8 μM) [25].

## 5. Excretion of Pyruvate by Halophilic and Non-Halophilic Prokaryotes

The rationale for the inclusion of pyruvate in the growth media that led to the isolation of *Hqr. walsbyi* was based on the fact that some other members of the *Halobacteria* excrete pyruvate when fed with sugars or with glycerol. This was first documented in *Halorubrum saccharovorum*, a carbohydrate-metabolizing member of the group [26]. When supplemented with glucose, *Hrr. saccharovorum* excretes acetic acid, D-lactic acid, and pyruvic acid [27]. The latter compound was identified using a colorimetric assay based on derivatization with 2,4-dinitrophenylhydrazine [28]. Formation of pyruvate was confirmed in a later study using HPLC. Pyruvate formation was then demonstrated in cultures of *Hrr. saccharovorum*, *Haloarcula marismortui*, and *Har. vallismortis* when supplemented with glycerol [13]. Excretion of pyruvate by halophilic Archaea fed with glycerol is not necessarily limited to cultures amended with excessively high glycerol concentrations: the formation of labeled pyruvate was documented in Dead Sea water inhabited by (mostly unidentified) Archaea following the addition of micromolar concentrations of ^14^C-glycerol [13].

In spite of the fact that pyruvate takes up a central position in the metabolism of aerobic heterotrophs and, therefore, pyruvate excretion is expected to be wasteful, there are quite a few reports of pyruvate being released by non-halophilic Bacteria of disparate phylogenetic affiliations. *E. coli* may release pyruvate to the medium when grown on succinate [29]. Marine luminescent bacteria grown on glucose can excrete up to half of the substrate carbon as pyruvate [30,31]. After depletion of the glucose, the pyruvate may be taken up again by the cells [30]. Mutants of *Alcaligenes eutrophus* impaired in the accumulation of poly (β-hydroxyalkanoate) can excrete pyruvate when fed with excess carbon (gluconate, lactate, or fructose) [32]. Pyruvate is also formed by *A. eutrophus* when supplied with increased concentrations of propionate [33]. Pyruvate (up to 0.57 mol/mol of glucose), together with lactate and acetate, were formed by a culture of *Vibrio natriegens* with glucose as the carbon and energy source [34]. Unbuffered cultures of *Bacillus cereus* grown on glucose-yeast extract medium excrete pyruvate [35]. *Streptomyces rimosus* grown on glucose and casamino acids releases pyruvate and α-ketoglutarate to the medium [36]. Pyruvate formation was also documented in *Streptomyces alboniger* [37].

## 6. Excretion of Dihydroxyacetone by Halophilic and Non-Halophilic Prokaryotes

Another three-carbon molecule that may play a central role in the community metabolism in hypersaline environments is dihydroxyacetone. Dihydroxyacetone was identified as an excretion product of the extremely halophilic bacterium *Salinibacter ruber* (*Bacteroidetes*) when supplemented with glycerol [38,39]. Analysis of the genome of *Hqr. walsbyi* showed the presence of a unique phosphoenolpyruvate-dependent phosphotransferase system involved in the phosphorylation of dihydroxyacetone, coupled with transport of the substrate through the membrane [21]. A similar transport system for dihydroxyacetone was identified in the genome of *Spiribacter* strain UAH-SP71. In one of the pathways annotated in this genome, glycerol is first metabolized to dihydroxyacetone and then phosphorylated by a dihydroxyacetone kinase to form dihydroxyacetone phosphate. Near this kinase gene, another gene was found coding for a phosphoenolpyruvate-dependent dihydroxyacetone phosphotransferase system. The enzyme encoded by this gene may be involved in the transport of dihydroxyacetone rather than in its catabolism [6].

There are also precedents for the excretion of dihydroxyacetone by non-halophilic bacteria. Thus, some acetic acid bacteria (*Gluconobacter*, *Acetobacter*) produce dihydroxyacetone in the presence of glycerol. *Gluconobacter oxydans* is even used for the commercial production of dihydroxyacetone [40,41].

## 7. Conclusions and Outlook

The above overview shows that two simple three-carbon compounds, pyruvate and dihydroxyacetone, may play key roles in the community metabolism in hypersaline ecosystems at or close to salt saturation. Both compounds can be formed from glycerol, a compound accumulated in molar concentrations within the cells of *Dunaliella salina* and other *Dunaliella* species, which are the main primary producers in high-salt environments. As glycerol produced by *Dunaliella* will eventually be released to the medium, it is postulated to be one of the main carbon and energy sources for heterotrophic halophilic microorganisms in saltern crystallizer ponds and many natural salt lakes. Understanding the interrelationships between different organisms metabolizing glycerol to incomplete oxidation products such as pyruvate and dihydroxyacetone on the one hand and other organisms that take up and further exploit these compounds on the other hand is highly relevant for the elucidation of the carbon and energy flow in the hypersaline ecosystems [42].

At present, we have no information about the *in situ* concentrations, rate of formation, and turnover times of pyruvate and other small metabolites such as dihydroxyacetone in saltern brines and in other high-salt aquatic systems. However, two decades ago, it was shown that the incomplete oxidation of glycerol to form lactate, acetate, and pyruvate was mediated by the Dead Sea microbial community following the addition of as little as 1.5–3 μM glycerol. These experiments showed that excretion of such partial oxidation products not only occurs at excessively high substrate concentrations but also at concentrations close to those expected to occur in the natural brines. Similar experiments in saltern crystallizer brine showed formation of lactate and acetate, but no pyruvate could be detected, possibly due to its rapid uptake and metabolism by other members of the community, notably *Haloquadratum*, which is abundantly found in such brines [13].

That glycerol, pyruvate, and dihydroxyacetone are preferred substrates for the mixed natural community of Archaea and Bacteria that inhabit saltern crystallizer brines can also be shown in simple experiments in which the community respiration is measured following the addition of relatively small (1 mM) concentrations of these compounds. The stimulating effect of glycerol on the community dark respiration of Eilat crystallizer brines was documented earlier [43]. Similar experiments in which the brine was amended with 1 mM Na-pyruvate or dihydroxyacetone resulted in similarly increased respiration rates (Oren *et al.*, unpublished results).

The advantage of pyruvate as a component of growth media for halophilic heterotrophic prokaryotes and the isolation of novel types of organisms, Archaea (*Haloquadratum*, *Halosimplex*), and Bacteria (*Spiribacter*) on such media shows that the importance of pyruvate as a key nutrient for halophilic prokaryotes is now well established. Some information on *in situ* concentrations and turnover rates of glycerol in hypersaline ecosystems was collected long ago [44], and additional experiments may supply more in-depth information on the role of glycerol as a nutrient in hypersaline brines. Similar information for pyruvate, as well as for dihydroxyacetone, is still completely lacking.
